# External Modulation Optical Coherent Domain Reflectometry with Long Measurement Range

**DOI:** 10.3390/s21165510

**Published:** 2021-08-16

**Authors:** Yinghong Xue, Yueping Niu, Shangqing Gong

**Affiliations:** School of Physics, East China University of Science and Technology, Meilong Road 130, Shanghai 200237, China; niuyp@ecust.edu.cn (Y.N.); sqgong@ecust.edu.cn (S.G.)

**Keywords:** optical fiber sensor, reflectometry, coherent detection, Rayleigh backscattering

## Abstract

Optical coherent domain reflectometry (OCDR) can achieve a high spatial resolution that is independent of the bandwidth of the receiver, but the measurement range is usually very limited. Here we propose an external modulation OCDR system, in which a pair of linear frequency-modulated pulses generated by one modulator are employed as the probe pulse and the reference, respectively. The spatial resolution is determined by the frequency modulation range of the pulse, and the measurement speed is boosted by orders because the proposed technology can simultaneously diagnose a section of fiber with each pair of pulses, while only a single point can be accessed at a time in typical OCDR. In the demonstrational experiment, a measurement range of up to 50 km is achieved with a spatial resolution of 1.4 m and a measuring time of less than 30 s.

## 1. Introduction

Optical reflectometry technologies, including optical time domain reflectometry (OTDR) [1,2,3,4], optical frequency domain reflectometry (OFDR) [5,6,7,8,9], and optical coherent domain reflectometry (OCDR) [10,11,12,13,14,15,16,17,18,19,20], have been widely used in the monitoring of fiber link and distributed sensing applications. The OTDR simply measures the round-trip time of reflection and Rayleigh backscattering (RBS) of the probe pulse. It has a long measurement distance of up to hundreds of kilometers, while the spatial resolution is usually on the meter level. Further improvement in the spatial resolution requires a narrower probe pulse width and a broader bandwidth of the receiver, which results in a poor signal-to-noise ratio and high system cost. In the OFDR system, the round-trip time of RBS is mapped to the beat frequency because of the linearly sweeping light source. It is famous for a high spatial resolution of up to micrometer level, but the maximum measurement range is limited by the nonlinearity of frequency sweeping and phase noise. Besides, the beat frequency is proportional to the length of the fiber under test (FUT), so a broadband receiver is necessary for a long sensing distance. Alternatively, OCDR has attracted a lot of attention because neither the spatial resolution nor the measurement range depends on the bandwidth of the receiver. Two types of OCDR technologies have been developed: optical low-coherence reflectometry (OLCR) [14] and the synthesis of the optical coherence function (SOCF) [15,16]. The OLCR uses a broadband light source, so it can only work at the 0th coherence spot. The measurement range of OLCR is determined by the mechanically adjustable range of the reference arm, which is very limited. Here we focus on SOCF–OCDR, in which a high-order coherent spot is adopted for sensing, and the accessing position is controlled via a modulation parameter. To simplify the configuration, the reference arm can be replaced by the Fresnel-reflected light of the FUT [17], or the optical frequency shifter can be removed [18]. An external modulation scheme using an electro-optic modulator (EOM) was also adopted to overcome the issues caused by internal modulation [19]. An acousto-optical modulator (AOM) was also adopted for frequency modulation with high sideband band suppression. In [20], a pair of AOMs is employed to modulate the probe and the local oscillator, respectively. However, the tunable range of the AOM cannot be fully adopted because of the necessary frequency offset between the probe and the local oscillator. An external modulation scheme was employed in the Brillouin OCDR system to extend the measurement range [21]. To guarantee that only one high-order coherent spot exists in the FUT, the typical SOCF–OCDR system consists of a long fiber delay line, which is usually several times longer than the length of the FUT. Although kilometer-order measurement range has been achieved [22], the size and insert loss are considerably large if the FUT is extended to tens of kilometers. In [23], external phase modulation is introduced to remove the periodic correlation peaks caused by using a comb source. A theoretical spatial resolution of 10.5 mm is realized over 100 m FUT, but the sensing position is scanned by the variable optical delay line, as is the case for OLCR. Besides, both OLCR and SOCF can only interrogate one position at a time, and the position moves sequentially or randomly along the FUT to diagnose the whole FUT. As a result, the measurement speed is relatively slow.

Here we propose an external modulation OCDR, in which the probe and the reference are generated via external frequency modulation. By digitally adjusting the time delay between pulses, only the first-order coherent spot exists in the system, and the long fiber delay line is avoided. The measurement range can be extended to tens of kilometers. Furthermore, if linear frequency modulation is adopted, the proposed system can interrogate a section of fiber simultaneously to boost the measurement speed. Compared with the OFDR system, the bandwidth of the receiver is independent of the length of the FUT. Compared with the system configuration in [20], the modulation bandwidth of the AOM can be fully used to achieve a higher spatial resolution, and the measurement speed is boosted by orders with section diagnosis of the fiber. In the demonstrational experiments, a 50 km long FUT can be diagnosed within 30 s.

## 2. Principle

**Figure 1:**
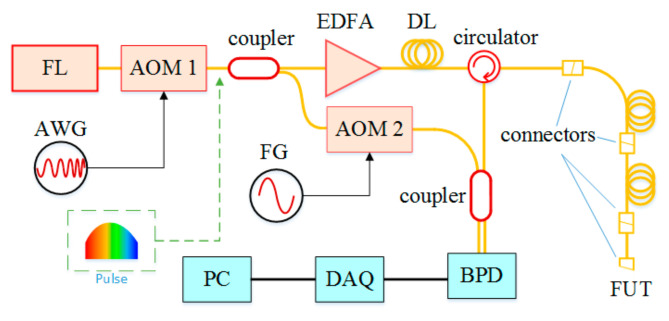
Systemic configuration. FL: fiber laser; AOM: acousto-optical modulator; AWG: arbitrary waveform generator; FG: function generator; EDFA: Er-doped fiber amplifier; DL: fiber delay line; FUT: fiber under test; BPD: balanced photo-detector; DAQ: data acquisition; PC: personal computer.

The systemic configuration is shown in Figure 1. The light wave from a continuous wave (CW) laser source is modulated by an acousto-optic modulator (AOM1). AOM1 works as both pulse generator and frequency shifter. It is driven by a sequence of linear frequency-modulated (LFM) radio frequency (RF) pulses from an arbitrary waveform generator (AWG). Each RF pulse has a time duration of *τ_a_* and a frequency chirp rate of *γ*. After passing by the AOM, the CW light wave is cut into pulses with the same frequency chirp rate of *γ*. Then the pulses are split into two beams by an optical coupler. One beam works as the reference, and its frequency is shifted by another AOM (AOM2). The other beam is injected into the FUT as the probe pulse after a fiber delay line (DL) and an Er-doped fiber amplifier (EDFA). The peak power of the probe pulse after the EDFA is about 200 mW. The RBS from the FUT is coherently detected with the reference on the balanced photo-detector (BPD), and the beat signal is sampled by a data acquisition (DAQ) device.

In each measurement, a pair of identical LFM pulses, P_1_ and P_2_, are applied to AOM1. The time interval *τ_d_* between the two pulses is controlled by the computer. It determines the accessing position on the FUT. The length of the DL is longer than the pulse width in fiber. So, at the time that the first pulse P_1_ in the probe path enters the FUT after passing by the DL, the corresponding pulse in the reference path has passed through the system. When the probe pulse P_1_ propagates in the FUT, its RBS as well as reflection by connectors of the FUT is led to the fiber coupler via the circulator.

The second probe pulse P_2_ is generated after the time interval *τ_d_*. It arrives at the BPD through the reference path, where it meets the RBS of the probe pulse P_1_. A beat signal is generated on the BPD, and it is collected by the DAQ device. The first pulse P_1_ in the reference path and the second pulse P_2_ in the probe path have no contribution to the measurement.

**Figure 2:**
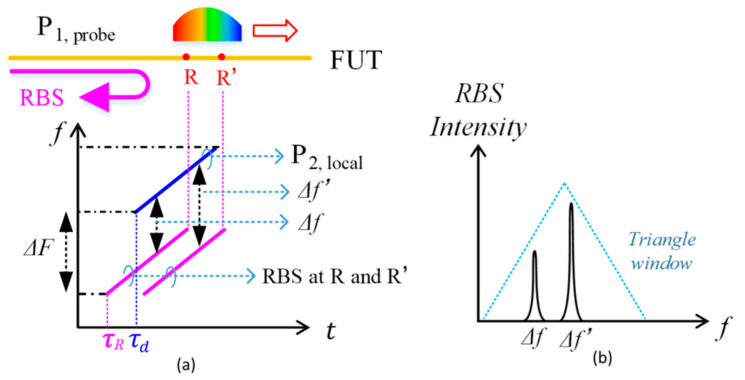
Principle of section access. (**a**) The RBS at positions R and R’ mix with the reference to generate beat frequencies; (**b**) the detected beat signal is applied by a triangle window.

Figure 2 shows the principle of detecting the RBS. The RBS of the probe pulse P_1_ occurs continuously along the FUT and sequentially arrives at the BPD. The RBS from any position also chirps within the time window *τ_a_*. It is coherently detected only if it meets the reference pulse P_2_ at the BPD. The reference pulse P_2_ is also a chirped pulse, but the frequency is shifted by Δ*F* because of AOM2, as shown in Figure 2a. Since both the RBS and the reference pulse are linearly chirped with an identical chirp rate γ, the optical frequency difference between the RBS at position R and the reference is constant, determined only by the relative time delay:

(1) $$\Delta f=\Delta F+\left(\tau_{R}-\tau_{d}\right)\gamma ,$$

where *τ_R_* is the round-trip time of RBS at position R. By choosing a proper frequency bias of Δ*F*, the beat frequency Δ*f* can be always positive, so the frequency Δ*f* can be simply obtained via Fourier transform. An equivalent configuration is the I/Q receiver instead of the AOM2. In this case, the beat frequency Δ*f* will have both positive and negative frequency components. They can be distinguished by I/Q demodulation technology with doubled PDs and DAQ channels [24,25,26].

Considering the RBS from a nearby position R’, the corresponding beat frequency is labeled as Δ*f’* in Figure 2. By detecting the spectrum of the beat frequency via Fourier transform, the RBS of position R and R’ can be simultaneously obtained. Since the duration of the probe pulse is *τ_a_*, the above coherent detection is valid when |*τ_R_*−*τ_d_*| < *τ_a_*. The RBS within a range of *L* = 2*τ_a_c*/*n* along the FUT can be detected simultaneously in each measurement in principle, where *c* is the velocity of light in vacuum, and *n* is the effective refractive index of the FUT. It should be noticed that the amplitude of the detected RBS is proportional to the overlap coefficient between the reference pulse and RBS, so the detected spectrum of the RBS is applied by a triangle window, as shown in Figure 2b. In practice, only the section of the spectrum near the center frequency (Δ*F*) is used for measurement. This section is normalized to remove the influence of the triangle window. The weak wings with low amplitude are filtered out because of the low signal-to-noise ratio.

The center of the section under diagnosis is determined by the time interval *τ_d_* between the pulses. Since *τ_d_* can be adjusted digitally, this system can diagnose any section of the FUT randomly. In contrast, typical SOCF can only diagnose one position of the FUT at a time. Besides, the length of the required fiber DL is only determined by the pulse length and is independent of the length of the FUT. A longer FUT can be effectively diagnosed by increasing the time interval *τ_d_* between the two pulses.

The spatial resolution of the proposed OCDR is determined by the spectrum resolution in the measurement of beat frequency and the mapping ratio of distance to beat frequency. The time window of measurement is the pulse duration of *τ_a_*, so the resolution of the spectrum is 1/*τ_a_* within the coherence length. With the chirp rate *γ* for the probe and reference pulses, the mapping coefficient from beat frequency to distance is calculated to be *c*/(2*nγ*). Finally, we obtain the expression of spatial resolution:

(2) $$\Delta l=\frac{c}{2\gamma n\tau_{a}}$$

where *γτ_a_* is the frequency chirping range of the probe pulse. According to Equation (2), a higher spatial resolution can be achieved by using a large frequency tuning range, which is the same as the spatial resolution of OFDR [27,28].

## 3. Results

### 3.1. System Setup

In the demonstrational experiments, the system configuration is the same as illustrated in Figure 1. A fiber laser (NKT, E15) at 1550.12 nm with 1 kHz linewidth is used as the optical source. The nominal frequency of the acousto-optic modulator (AOM1) is 200 MHz. It is driven by an arbitrary waveform generator (Tektronix, AFG3252C), which generates a frequency chirping RF pulse with a time duration of 8 μs. The frequency chirp range of the RF pulse is 160 MHz to 240 MHz. The transmission of the AOM decreases as the frequency of the driving RF signal deviates from the nominal frequency. The full width at half maximum (FWHM) of the frequency sweeping range is about 70 MHz, corresponding to a theoretical spatial resolution of 1.4 m in fiber according to Equation (2). AOM2 on the reference path works as a frequency shifter with a driving frequency of 80 MHz. A balanced photo-detector (BPD, Thorlabs, PDB480C) is used to receive the mixed light wave. A data acquisition (DAQ) device (NI, 5185) with a sampling rate of 520 MSa/s is used to collect the beat signal. The delay line (DL) is 2 km long, which is slightly longer than the probe pulse duration in fiber but much shorter than the FUT. The total length of FUT is about 50 km, composed by two 25 km long spools of fiber. A 3 m fiber patch cord is attached at the far end of the FUT.

According to the frequency configuration of the two AOMs, the frequency shift of the probe pulse is from 160 MHz to 240 MHz, while that of the reference pulse is from 240 MHz to 320 MHz. So, the beat frequency detected by the BPD covers a range of 0–160 MHz. To guarantee a high enough signal-to-noise ratio, only the frequency components around 80 MHz are utilized in the measurement. The frequency range of the utilized signal determines the range of accessing section length in one measurement (one pair of pulses).

### 3.2. Experimental Results

To diagnose the whole FUT, the pulse interval *τ_d_* varies from 10 μs to 550 μs with a step of 500 ns. With the help of a synchronizing system, the DAQ device only works in the duration of the reference pulse, so as to guarantee coherent detection. Fast Fourier transform is applied to the collected data in each measurement (one pair of RF pulses sent to AOM1), and the power spectral density in the range of 77.5 MHz to 82.5 MHz is selected and normalized to compensate for the window effect, corresponding to an overlap time of no less than 7.75 μs (over 8 μs) between the reference and the RBS. Each measurement covers a length of 50 m along the FUT. Considering the theoretical spatial resolution of 1.4 m, each measurement can diagnose 36 positions simultaneously. At each time interval, the pulse pairs repeat for 100 times to achieve a better signal-to-noise ratio via average. By rebuilding the data of different time intervals in sequence, the RBS of the whole FUT is obtained in less than 30 s.

**Figure 3:**
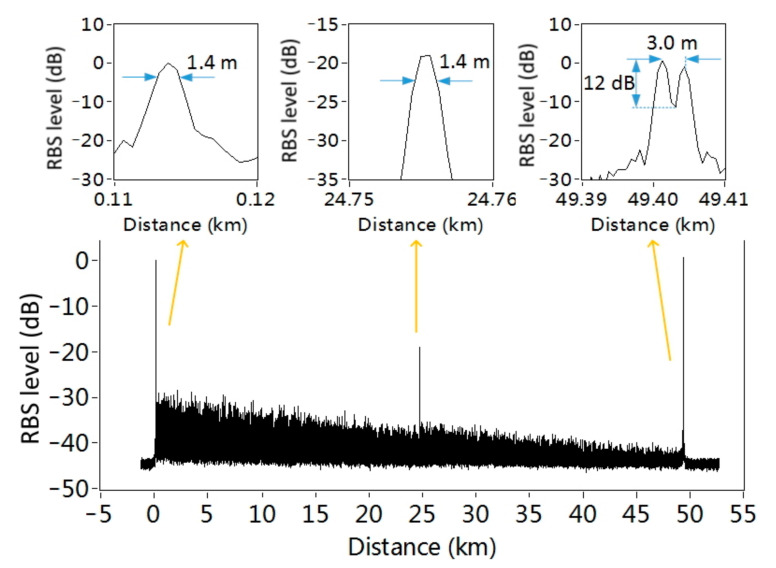
The normalized intensity of Rayleigh backscattering (RBS) as well as the reflection of connectors along the 50 km FUT after 100 averages. Insets: the reflection of connectors at the near, middle, and far end of the FUT.

The obtained RBS trace of the FUT is shown in Figure 3. The large fluctuation of the RBS amplitude is caused by the coherence fading, because the narrow linewidth fiber laser keeps constant wavelength during the whole experiment. The Fresnel reflections at the connectors along the FUT are clearly observed, which can be used to evaluate the spatial resolution of the proposed system. According to these reflection peaks, the spatial resolution is 1.4 m at both the beginning and the middle of the FUT, which is the same as the theoretical spatial resolution calculated from the FWHM of the probe pulse spectrum. At the far end of the FUT, the two connectors of the 3 m bench cord are clearly separated, with a notch depth of 12 dB. The spatial resolution is almost the same along the whole length of FUT, proving that the proposed technique has a large measurement range without degeneration in spatial resolution. To the best of our knowledge, this is the longest sensing distance using the SOCF–OCDR configuration.

In the traditional SOCF–OCDR system, the frequency tuning rate is a sinusoidal waveform. Except for the highly coherent spots, the frequency difference between the reference and the RBS of the probe is not constant at other positions. So, the system can only diagnose the highly coherent spot. In the proposed method, if linear frequency modulation is adopted, there will be a linear mapping of the beat frequency to the position of RBS. As a result, the system can diagnose a section of the FUT at a time. Besides, although the probe and reference pulse are generated by the same modulator (AOM1 in the demo configuration), the RBS of the probe is coherently detected with the next reference pulse. The proposed system actually utilizes the first-order coherent spot. The short delay line in the configuration avoids the interference of the unwanted pulses.

### 3.3. Discussion

Although in the demonstrational experiment, a high sampling rate device DAQ is adopted, the valid bandwidth is only 5 MHz, so a minimum sampling rate of 10 MSa/s is possible for the DAQ device if a proper bandpass filter and under-sample technology are adopted. If the I/Q demodulation configuration is adopted, the required bandwidth can be reduced to a half of 5 MHz. Besides, the bandwidth is proportional to the detection range of the FUT. An even narrower band DAQ device is also acceptable at the cost of a smaller detection range of the FUT without degeneration of the spatial resolution. In the extreme case that the bandwidth of the DAQ tends to zero, the proposed system can only detect one position of the FUT, which is the case for the typical OCDR system.

Due to the limited frequency shifting range of the AOM, the spatial resolution is meter level in the demonstrational experiments. It can be improved if broader band modulators such as EOM are employed, as in the configuration in [13]. The sensing distance is independent of spatial resolution, and the demonstrated 50 km length is much longer than that for current OCDR systems. Generally, the frequency tuning range of external modulation is smaller than that of internal modulation, so the proposed method is suitable for long-distance sensing with moderate spatial resolution and a narrow-bandwidth receiver.

To guarantee the spatial resolution described in Equation (2), the coherent length of the laser source should be at least two times longer than the length of the FUT. If the length of the FUT is far beyond the above limitation, the resolution of the detected beat frequency broaden by 2*δf*, where *δf* is the linewidth of the laser [29]. In this case, the spatial resolution will degenerate to *c*/2*nγτ_a_* + *cδf*/*nγ*.

Although the proposed system is very similar to the OFDR system, it has a key difference. In the typical OFDR system, the beat frequency is proportional to the time delay of the RBS from the FUT, so the beat frequency will increase linearly as the length of the FUT increases. In the proposed system, the local oscillator is a time delayed copy of the probe pulse with frequency shift. The adjustable time delay of the local oscillator compensates for the time delay of the RBS, so the beat frequency can be maintained in a moderate frequency range, and it is independent of the length of the FUT.

Traditional OTDR and coherent OTDR (COTDR) can also achieve meter-level spatial resolution over tens of kilometers of fiber. However, the same resolution of 1.4 m will require a very narrow probe pulse duration of 14 ns and a broadband receiver no smaller than 70 MHz. Such a narrow pulse width will result in weak Rayleigh backscattering and thus a poor signal-to-noise ratio. The time-gated digital optical frequency domain reflectometry (TGD-OFDR) system solves the trade-off between pulse duration and spatial resolution by using linear frequency-modulated probe pulses [30], but the bandwidth of the receiver should also be equal to the frequency sweeping range of the probe pulse (70 MHz if the spatial resolution is 1.4 m), and it is much higher than that of the proposed OCDR system with the same spatial resolution.

## 4. Conclusions

Using external linear frequency modulation and dual pulse interrogation configuration, we propose an OCDR system with a long measurement range and the capability of section access. A long fiber delay line is avoided by the digital control of the time interval between pulses. In the demonstrational experiments, a spatial resolution of 1.4 m was obtained over 50 km FUT. The proposed OCDR technique can realize a long measurement range with a moderate spatial resolution and a short measurement time, making it very useful for long-distance applications.

## Data Availability

The data presented in this study are available on request from the corresponding author.
